# Reactive Oxygen Species in Cystic Kidney Disease

**DOI:** 10.3390/antiox13101186

**Published:** 2024-09-30

**Authors:** Sanat Subhash, Sonya Vijayvargiya, Aetan Parmar, Jazlyn Sandhu, Jabrina Simmons, Rupesh Raina

**Affiliations:** 1 Department of Internal Medicine, Northeast Ohio Medical University, Rootstown, OH 44272, USA; ssubhash@neomed.edu (S.S.); jsimmons1@neomed.edu (J.S.); 2 Department of Internal Medicine, Emory University School of Medicine, Atlanta, GA 30322, USA; sonya.vijayvargiya@emory.edu; 3 Akron Nephrology Associates, Cleveland Clinic Akron General Medical Center, Akron, OH 44307, USA; parmar.57@buckeyemail.osu.edu (A.P.); jsandhu57@susdgapps.org (J.S.)

**Keywords:** cystic kidney disease (CKD), oxidative stress, tolvaptan, ADPKD (autosomal dominant polycystic kidney disease), mitochondrial antioxidants

## Abstract

Polycystic kidney disease (PKD) is a rare but significant renal condition with major implications for global acute and chronic patient care. Oxidative stress and reactive oxygen species (ROS) can significantly alter its pathophysiology, clinical outcomes, and treatment, contributing to negative outcomes, including hypertension, chronic kidney disease, and kidney failure. Inflammation from ROS and existing cysts propagate the generation and accumulation of ROS, exacerbating kidney injury, pro-fibrotic signaling cascades, and interstitial fibrosis. Early identification and prevention of oxidative stress and ROS can contribute to reduced cystic kidney disease progression and improved longitudinal patient outcomes. Increased research regarding biomarkers, the pathophysiology of oxidative stress, and novel therapeutic interventions alongside the creation of comprehensive guidelines establishing methods of assessment, monitoring, and intervention for oxidative stress in cystic kidney disease patients is imperative to standardize clinical practice and improve patient outcomes. The integration of artificial intelligence (AI), genetic editing, and genome sequencing could further improve the early detection and management of cystic kidney disease and mitigate adverse patient outcomes. In this review, we aim to comprehensively assess the multifactorial role of ROS in cystic kidney disease, analyzing its pathophysiology, clinical outcomes, treatment interventions, clinical trials, animal models, and future directions for patient care.

## 1. Introduction 

Polycystic kidney disease (PKD) is a rare but significant renal condition with major implications for global acute and chronic patient care. Oxidative stress can alter its pathophysiology, clinical outcomes, and treatment. Cystic kidney disease (CKD) refers to the occurrence of kidney cysts in adult and pediatric patients, with varying clinical characteristics and risk factors [1]. The etiology of CKD can be inherited or non-inherited, with inherited autosomal dominant polycystic kidney disease (ADPKD) affecting nearly 10% of dialysis patients and contributing to adult end-stage renal disease (ESRD) [2,3]. Other uncommon but notable inherited conditions, including autosomal recessive polycystic kidney disease (ARPKD), glomerulocystic kidney disease, nephronophthisis, and autosomal dominant tubulointerstitial kidney disease, also exhibit similar clinical manifestations and are associated with an elevated risk of developing disease conditions like diabetes and gout [1]. Oxidative stress can have a significant negative impact on renal health, particularly in the development of cystic kidney disease and its extrarenal manifestations, such as atherosclerosis, endothelial injury, and cardiovascular disease [4]. Oxidative stress arises from a cellular imbalance toward increased oxidation, stemming from reactive oxygen species (ROS), damaging tissues, inducing inflammation, and exacerbating the progression of cystic kidney disease [4]. In ADPKD, extrarenal manifestations of hypertension and endothelial dysfunction are connected to endothelial NOS (nitric oxide synthase) uncoupled due to increased ROS production [5]. Considering the impact of ROS on cystic kidney disease progression, determining pathways for enhanced prevention and treatment, and critically evaluating clinical research are vital for nephrologists and healthcare workers supporting CKD patients.

In this review, we aim to comprehensively assess the multifactorial role of ROS in cystic kidney disease, analyzing its pathophysiology, clinical outcomes, treatment interventions, clinical trials, animal models, and future directions for patient care.

## 2. Pathophysiology

**Figure 1:**
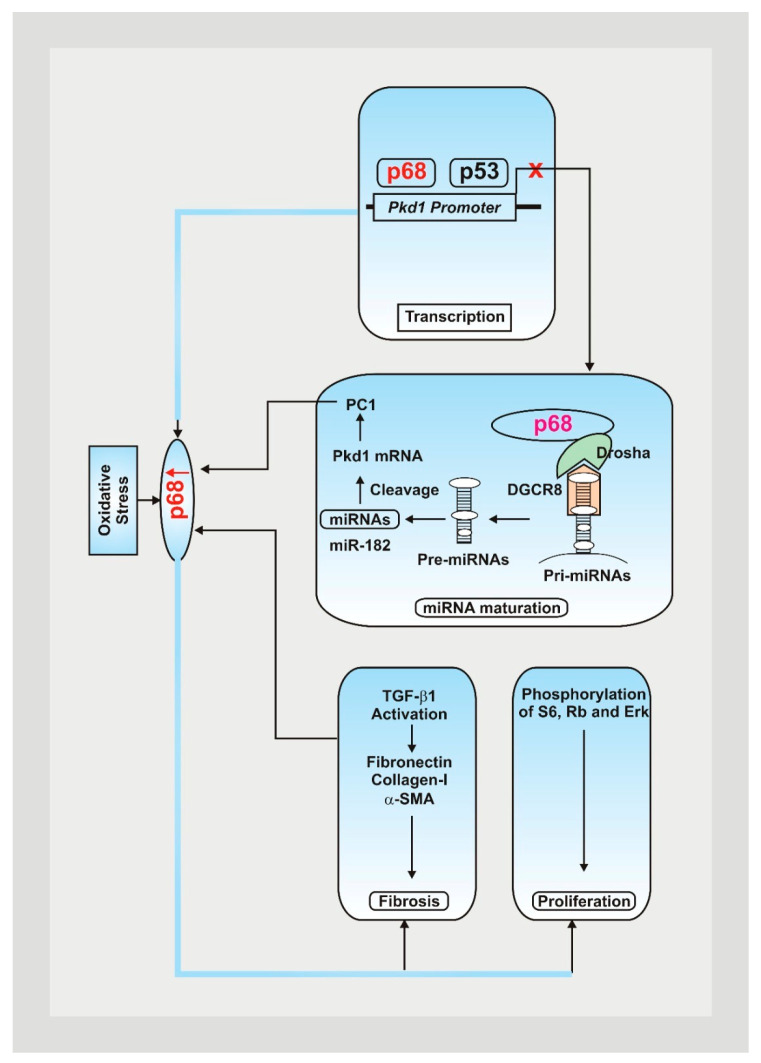
Mutations within the Pkd1 genes play a significant role in the pathogenesis of ADPKD, contributing to cyst formation, issues with DNA damage pathways, and cellular disruptions [7,8]. Oxidative stress contributes to an increase in p68, decreasing Pkd1 gene expression by attaching to the Pkd1 promoter and upregulating the expression of PKD-associated miRNAs, resulting in posttranscriptional cleavage and loss of Pkd1 mRNA [9]. Fibrotic markers and PKD signaling pathways are also upregulated, leading to a rise in cystic renal epithelial cell proliferation and resulting fibrosis in ADPKD kidneys [9].

Cystic kidney disease can be characterized by its inheritance pattern. ARPKD is rarer and more severe than ADPKD due to a mutated form resulting from a mutation in the PKHD1 gene [6]. Along with polycystic kidneys, individuals with ARPKD also develop hepatic fibrosis, making them more prone to death at a young age due to respiratory failure [6]. Most PKD is characterized by ADPKD, which results from mutations in the PKD1 and PKD2 genes [6]. Patients typically develop symptoms such as flank pain between the third and fifth decade of life, with most individuals presenting with PKD1 mutations (85%). The PKD1 phenotype has also been associated with additional renal cysts and progressively evolves into renal failure, as demonstrated in Figure 1 [6].

PKD1 and PKD2 both code for the polycystin protein, which responds to cell oxygen levels and is thereby a product that regulates mitochondrial function and cell metabolism. Polycystins are receptor channels in the cilia of epithelial cells, which increase intracellular calcium for kidney tubular formation [10,11].

In PKD, defects in the cilia disrupt cilia-signaling pathways, including calcium balance, Hedgehog, Wnt/B-catenin, and cyclic adenosine monophosphate (cAMP), contributing to cyst formation [6]. In particular, changes in calcium regulation lead to increased intracellular cAMP and high chloride-rich fluid secretion, resulting in aberrant proliferation, increased growth factors, and cystogenesis [6,10,11].

**Figure 2:**
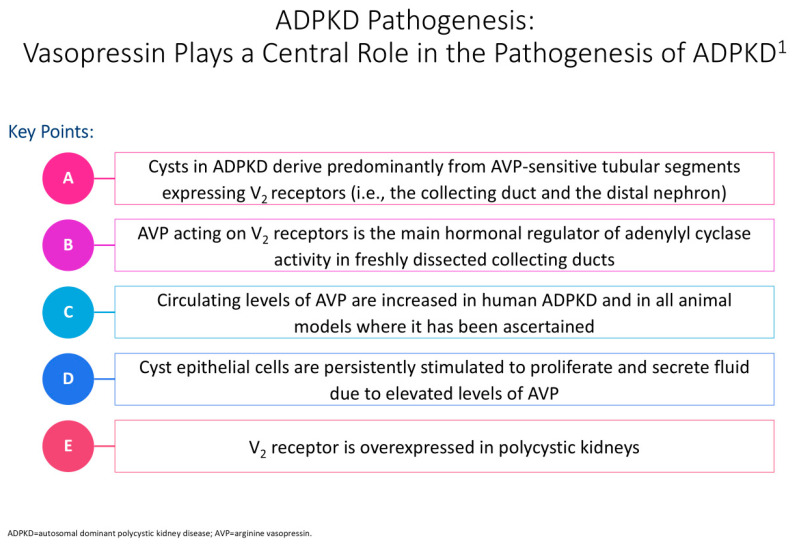
Cyclic adenosine monophosphate (cAMP) plays a significant role in the pathogenesis of autosomal dominant polycystic kidney disease (ADPKD) in epithelial cells [12]. The cyst is shown in the tubular cell lining, and the PC1 and PC2 complex regulates calcium levels after primary stimuli sensing at the apical pole [12]. Issues with this complex can contribute to modified intracellular Ca2þ levels, with an increased cAMP concentration linked to a decrease in intracellular calcium levels [10]. This rise in cAMP levels further contributes to protein kinase A(PKA)-mediated phosphorylation of pathway mediators, resulting in issues with flow sensing, tubulogenesis, chloride channel cystic fibrosis transmembrane conductance regulator (CFTR)-driven transepithelial fluid secretion, a rise in water channels, and additional transcriptional regulation of cell proliferation factors [10]. In PKD, defects in cilia disrupt cilia-signaling pathways, including calcium balance, Hedgehog, Wnt/B-catenin, and cyclic adenosine monophosphate (cAMP), contributing to cyst formation [6]. In particular, changes in calcium regulation lead to increased intracellular cAMP and high chloride-rich fluid secretion, resulting in aberrant proliferation, increased growth factors, and cystogenesis [6,10,11]. The superscript “1” is meant to cite Ref. [12].

Figure 2 illustrates the impact of vasopressin on the pathogenesis of polycystic kidney disease. Halvorson et al. describe the interaction between cilia disruption and planar cell polarity (PCP), stating how PCP is necessary for organogenesis, and that in PKD, random patterns of cell division result in tubular dilation and cystogenesis [6].

Dysregulated cell metabolism and function in the setting of mitochondrial oxidative stress is the primary pathophysiology behind PKD [11]. ROS from dysfunctional mitochondria have been implicated in cyst formation in PKD, and ROS levels in cells may positively correlate with disease severity [11]. The generation of ROS results in the dysregulation of multiple pathways, including mitochondrial apoptosis, NADPH oxidase and xanthine oxidase activity, and cell inflammation.

The primary source of ROS generation in renal tubular cells and endothelial cells in PKD is NADPH-oxidase complex-4 (NOX4) [13]. Early PKD is often associated with increased NADPH oxidase activity and upregulation of NOX4 expression in kidney cells, which generate free radicals and ROS, inducing oxidative stress and endothelial dysfunction [13].

In addition, Korsmo, Ekperikpe, and Daehn highlight that patients with kidney renal diseases including PKD have increased xanthine oxidoreductase (XOR) levels [14]. XOR results in hyperuricemia, which causes tubular injury and a pro-inflammatory state; it also induces NOX4 and the production of ROS. Finally, XOR inhibits nitric oxide (NO) synthase in endothelial cells and nitric oxide release, resulting in low levels of NO and vascular manifestations of PKD.

On a cellular level, moderate ROS levels act as important vital signaling molecules that control cell proliferation, differentiation, and death [15].

However, excess generation of ROS, such as in PKD, causes the peroxidation of mitochondrial DNA, proteins, and lipids, impaired cell function and signaling, alteration of cell ATP production, and increased expression of pro-fibrotic factors. This results in autophagy, hypoxia, and apoptosis [10]. Highly elevated ROS levels can also uncouple endothelial NO synthase, further exacerbating the oxidative stress produced by XOR and extra-renal manifestations of PKD [5]. Table 1 provides an overview of key signaling cascades impacted in PKD.

Mitophagy, a protective form of autophagy that reconstructs mitochondria, is also increased in PKD to clear mitochondria damaged by oxidative stress and regenerate kidney integrity [10]. This overactive process likely causes cell energy depletion, thus resulting in an imbalance of apoptosis and a chronic state of inflammation in mitochondrial kidney renal cells [10].

Inflammation from ROS and existing cysts further propagate the generation and accumulation of ROS, contributing to the exacerbation of kidney injury, pro-fibrotic signaling cascades, interstitial fibrosis, and the eventual impairment of renal function and CKD [10,13].

## 3. Clinical Outcomes

Hypertension is an early clinical outcome of ADPKD, typically observed before the decline in renal function and the glomerular filtration rate (GFR) [5]. In total, 50% of patients between the ages of 24 and 30 years and 100% of patients with renal failure develop hypertension. Menon et al. detailed that oxidative stress markers were elevated in early-stage ADPKD, preceding the onset of clinically significant hypertension. Oxidative stress markers did not worsen or change with progressive renal disease [16].

The study also demonstrated that hypertensive patients with PKD developed end-stage renal disease (ESRD) at an accelerated pace and that oxidative stress and PKD clinical outcomes may be connected.

Hypertension is a well-known risk factor for cardiovascular morbidity and mortality and is associated with the worsening of existing renal disease, including in those with PKD [5]. Excessive ROS generation and NO depletion in PKD cause endothelial dysfunction and tissue hypoxia. This, in combination with multiple kidney cysts compressing the renal vasculature and causing ischemia, activates the renin–angiotensin–aldosterone system (RAAS), promoting high blood pressure and increasing premature cardiovascular complications, such as left ventricular hypertrophy (LVH). As a result, early blood pressure control is key to PKD management. Ecder and Schrier emphasize that cardiovascular involvement in ADPKD begins at an early age; patients often present with manifestations such as diastolic ventricular dysfunction and valvular defects due to endothelial dysfunction causing aberrant blood flow [17].

Hypertension is not the only clinical outcome of PKD. Like the mechanism that underlies the development of hypertension and cardiovascular complications, those with PKD are at significantly higher risk for chronic kidney disease and renal failure [18]. Expanding cysts displace kidney tubules and obstruct renal blood flow, worsening the tissue hypoxia, oxidative stress, and inflammation generated by ROS. The resulting apoptosis of mitochondria and fibrosis of kidney cells leads to a loss of functioning nephrons and irreversible loss of renal function. Patients with PKD often experience decades of chronic flank pain, hematuria, and renal cyst infections. Ultimately, approximately half of patients develop ESRD by age 60, with ADPKD accounting for 2.5% of all ESRD cases [6,19]. In ADPKD, approximately 50% of patients require dialysis and renal transplantation by age 70 [6].

## 4. Treatment and Therapeutic Interventions

Oxidative stress has treatments ranging from direct antioxidant therapies to combination drug therapies, and the early identification and prevention of oxidative stress and ROS can contribute to reduced cystic kidney disease progression and improved longitudinal patient outcomes. Treatments for oxidative stress range from direct antioxidant therapies to combination drug therapies.

Established biomarkers, such as advanced oxidation protein products (AOPPs), uric acid (UA), and ferric-reducing ability of plasma (FRAP), can be utilized within a panel to measure ROS levels in kidney disease patients [4,20]. However, these urine or saliva biomarkers are subject to significant interpatient variability, posing issues for broader implementation in clinical practice [21].

To counter this, NOX4 is a significant producer of ROS within renal tubular epithelial cells (TECs) and endothelial cells (ECs) in patients with early stages of ADPKD [13]. Overexpression of ROS by NOX4 can cause mitochondrial abnormalities in endothelial cells, leading to endothelial dysfunction, higher mitochondrial protein oxidation, vascular complications, and accelerated progression of renal disease [13]. An experimental analysis by Bernard et al. analyzing the impact of NOX4 in lung fibroblasts reported that NOX4 significantly suppressed mitochondrial biogenesis and metabolism, and inhibition through genetic or pharmacological methods reversed these effects [22]. Thus, inhibition of NOX4 can assist in reducing ROS through the elevation of mitochondrial biogenesis and metabolism.

Mitochondria produce nearly 90% of the body’s ROS during mitochondrial metabolism, with renal antioxidant systems mitigating this creation under normal physiological conditions [23,24].

In cases of increased ROS production resulting in oxidative stress in cystic kidney disease patients, additional mitochondrial antioxidants can work to protect mitochondrial function and reduce cellular damage [23,25].

The application of the mitochondrial antioxidant MitoTEMPO[(2-(2,2,6,6-Tetramethylpiperidin-1-oxyl-4-ylamino)-2-oxoethyl)triphenylphosphoniumchloride monohydrate] in sepsis-induced acute kidney injury (SAKI) rodents reported improved mitochondrial function, microcirculatory perfusion, renal function, and long-term survival outcomes [22]. Another animal study by Daneshgar et al. noted that a mitochondrial-protective tetrapeptide treatment (SS31) hindered ADPKD-like disease progression, decreased reduced mitochondrial ROS, and prevented obstructed further oxidative harm [26]. While there are no human clinical studies analyzing the impact of mitochondrial antioxidants on ameliorating cystic kidney diseases, animal research in kidney disease shows promising results for mitigating oxidative stress and preserving renal function.

In addition, increased serum uric acid levels and uric acid supersaturation have been proposed as contributing factors to ADPKD progression, the incidence of ESRD, and the age of onset for hypertension [27,28]. Xanthine oxidase inhibitors (XOis) are provided for the treatment of hyperuricemia in renal conditions such as chronic kidney disease, working to decrease circulating uric acid levels, lower oxidative stress, and inhibit glomerular hypertension [29].

A study on genetically modified polycystic kidney disease (PKD) mice by Chaudary et al. reported that treatment with oxypurinol, a xanthine oxidase inhibitor, decreased rates of cyst formation in the Pkd1 mice model [30].

However, a prospective study by Brosnahan et al. of 671 PKD patients stratified by uric acid tertiles found no association between raised uric acid levels and ADPKD disease progression [31]. Further randomized controlled trials are required to accurately assess the relationship between uric acid and cystic kidney disease and evaluate the longitudinal impact of XOis treatment.

Furthermore, dietary, nutritional, and lifestyle modifications can also play a significant role in combating oxidative stress in cystic kidney disease and improving patient outcomes [32]. The administration of exogenous ketone b-hydroxybutyrate (BHB) in rats was shown to suppress PKD progression, and BHB has also been linked to lowered oxidative stress [33,34]. Diets involving fasting or calorie restriction, including time-restricted feeding (TRF), a ketogenic diet, and acute fasting all resulted in elevated ketone BHB levels in mouse models [33]. Small pilot trials have been conducted to measure the feasibility of ketogenic diets for ADPKD patients; however, there remains a need for a large-scale, comprehensive trial evaluating the impacts of TRF, a ketogenic diet, exogenous ketone BHB, and acute fasting on ADPKD progression and oxidative stress reduction [35,36,37].

Currently, tolvaptan remains the singular approved disease-modifying drug available for treating ADPKD; it has been shown to decrease total kidney volume (TKV), reduce the rate of kidney function decline, and relieve oxidative stress [38,39].

A comparative analysis by Rigato et al. of 18 ADPKD patients stated that tolvaptan treatment decreased oxidative stress-signaling proteins and provided additional protection against oxidative stress, with the untreated group reporting significantly higher rates of the oxidative stress biomarkers p22^phox^ and MYPT-1 phosphorylation state [40].

Tolvaptan was also found to activate the nuclear factor erythroid 2-related factor 2 (Nrf2) signaling pathway, increasing the responsiveness and production of antioxidants in reaction to oxidative stress [41]. Polyuria, nocturia, hepatotoxicity, and hypertension are major side effects of tolvaptan treatment reported across clinical patient trials. It is recommended that providers carefully monitor for these adverse effects and balance the risks and benefits during treatment [39].

Table 2 illustrates major studies and outcomes for the various therapeutic interventions targeting ROS in cystic kidney disease.

Unfortunately, ARPKD, glomerulocystic kidney disease, nephronophthisis, and autosomal dominant tubulointerstitial kidney disease do not have established primary disease-modifying drugs available and require comprehensive treatment strategies focused on symptom management and supportive care.

## 5. Future Directions

Cystic kidney diseases, though uncommon, present a significant risk for ESRD and other adverse patient outcomes, such as cardiovascular disease. There remains a critical need to increase research regarding biomarkers, methods for early detection, the pathophysiology of oxidative stress, novel therapeutic interventions, and randomized clinical trials.

For many treatment methods outlined above, including dietary modifications, NADPH oxidase inhibitors, mitochondrial antioxidants, and xanthine oxidase inhibitors, randomized clinical trials have not been conducted in human cystic kidney disease patients. Additionally, more research is available for ADPKD compared with ARPKD, glomerulocystic kidney disease, nephronophthisis, and autosomal dominant tubulointerstitial kidney disease.

There are limited available studies discussing sources or treatment of ROS for ARPKD, glomerulocystic kidney disease, nephronophthisis, and autosomal dominant tubulointerstitial kidney disease, indicating a major gap in the understanding of the pathophysiology and treatment methods for these conditions. Research focusing on individual treatments for these conditions and reviews analyzing specific outcomes are vital next steps to accurately assess patient care and improve treatment strategies.

In addition, combination therapy regarding the impact of ROS in cystic kidney disease is another unexplored area, and clinical trials and systematic reviews integrating pharmacological and lifestyle treatment modalities could help create more comprehensive management strategies. Currently, no established guidelines or protocols are available for clinicians to assess and manage ROS in cystic kidney disease patients.

Oxidative stress could be a significant biomarker in PKD disease diagnosis and progression. For example, higher levels of oxidized LDL cholesterol, MDA (malondialdehyde), and ADMA (asymmetric dimethyl arginine) and lower levels of SOD (superoxide dismutase) have been observed in PKD patients.

Creating comprehensive guidelines establishing methods of assessment, monitoring, and intervention for oxidative stress in cystic kidney disease patients is imperative to standardize clinical practice and improve patient outcomes.

Furthermore, artificial intelligence (AI) presents an incredible opportunity for researchers and clinicians in cystic kidney disease to increase the speed of data analysis, improve patient management, and enhance clinical outcome assessment [48].

Machine learning (ML) and deep learning (DL) models have been used for cyst detection, TKV measurement, the predictive modeling of ADPKD progression, and providing ADPKD patient prescriptions for tolvaptan [49,50,51]. AI models can also reduce the costs of statistical analysis and trial creation, assisting researchers in conducting rare cystic disease studies.

**Figure 3:**
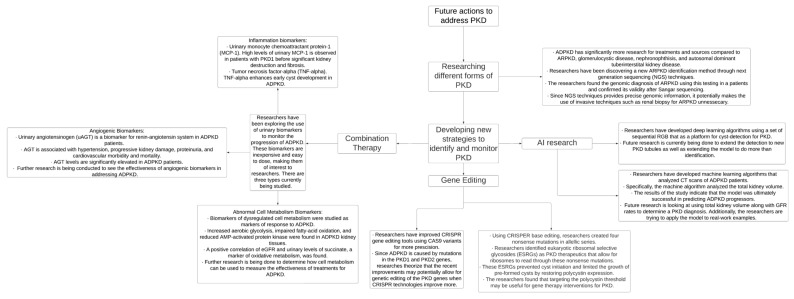
PKD—polycystic kidney disease, AI—artificial intelligence, CRISPR—clustered regularly interspaced short palindromic repeats, CT—computed tomography, and ADPKD—autosomal dominant polycystic kidney disease [49,50,52,53,54,55].

Finally, modern technologies involving genetic editing and genome sequencing could allow researchers to address mutations that cause inherited cystic kidney disease, promoting enhanced diagnostic and treatment capabilities, and these advancements are illustrated in Figure 3. By increasing preventative care, conducting randomized controlled trials, and creating systematic guidelines, providers can improve the early detection and management of cystic kidney disease and mitigate adverse patient outcomes.

## 6. Conclusions

Excess production of ROS in PKD results in changes in cell metabolism and signaling, contributing to cystogenesis. Dysregulated mitochondrial apoptosis, increased activity of NOX4 and XOR, and low levels of NO not only result from ROS production but also stimulate greater ROS production, leading to inflammation, tubular injury, renal fibrosis, and endothelial injury. Hence, ROS can be associated with the clinical complications of PKD, most notably ESRD and cardiovascular disease. AOPPs, UA, and FRAP are common biomarkers for measuring ROS levels in cystic kidney disease patients.

However, additional research needs to be conducted regarding early detection methods. NOX4 and xanthine oxidase inhibitors, mitochondrial antioxidants, dietary modifications, and tolvaptan are potential treatments addressing ROS in cystic kidney disease, yet there is limited randomized controlled trial research performed on humans demonstrating their efficacy and safety.

Integrating modern genetic and AI technology alongside combination therapy and clinical guidelines for ROS management, prevention, and treatment in cystic kidney disease could improve the quality and longevity of life for affected patients.

## Figures and Tables

**Table 1:** Summary of key signaling cascades impacted in PKD.

Key Signaling Cascade	Renal and Extra-Renal Consequences
NOX4	NOX4 is the primary source of ROS generation in renal cells and endothelial cells. Upregulation of NOX4 in PKD generates ROS, causing oxidative stress and endothelial dysfunction.
XOR	Increased XOR activity in PKD causes hyperuricemia, induction of NOX4, and inhibition of NO synthase. This results in inflammation, ROS production, and endothelial injury.
NO synthase	Surplus ROS uncouples NO synthase, and increased XOR inhibits NO synthase. Both deplete the NO supply, causing oxidative stress and vascular damage.
Mitochondrial apoptosis	Excess ROS oxidizes mitochondrial DNA, proteins, and lipids. It impairs cell function and signaling and ATP production, resulting in hypoxia, overactive mitophagy, apoptosis, fibrosis, and inflammation.

NOX4 (NADPH oxidase 4), ROS (reactive oxygen species), PKD (polycystic kidney disease), XOR (xanthine oxidoreductase), NO synthase (nitric oxide synthase), and ATP (adenosine triphosphate).

**Table 2:** Outcomes and mechanisms of action for drug treatments for PKD.

Medication	Author	Year	Methods	Outcomes	Mechanism of Action
Tolvaptan	Calvaruso et al. [38]	2023	Mayo Clinic Imaging Class (MCIC) and total kidney volume measurements were taken for 523 patients of 18 years or older with confirmed ADPKD to compare the properties of patients undergoing treatment versus patients not undergoing tolvaptan treatment.	In total, 60% (315/523) of patients with ADPKD were considered to be at high risk of progressing to ESKD, but only 30% (96/315) of them were treated with tolvaptan at their respective follow-ups.	Tolvaptan is a competitive antagonist at the V2 vasopressin receptor in the renal collecting ducts. As a result, aquaporin synthesis and transport are improved, leading to better water retention while reducing plasma osmolality. As a result, urine volume is reduced but urinary sodium ion secretion continues [42].
	Raina et al. [39]	2022	The research conducted a systematic review of 22 pieces of literature to determine the side effects, efficacy, and complications of tolvaptan use for ADPKD.	TEMPO 3:4 and REPRISE trials showed a change in eGFR from pre-treatment baseline to post-treatment of 1 mL/min/1.73 and 1.3 mL/min/1.73, respectively, for patients undergoing ADPKD treated with tolvaptan. There was a mean decrease of 49% in total kidney volume from baseline to post-treatment in the TEMPO 3:4 study.	
	Rigato et al. [40]	2022	The research examined the OxSt of 27 patients aged 18–65 through six tests: mononuclear cell p22phox protein expression, NADPH oxidase key subunit, MYPT-1 phosphorylation state, a marker of Rho kinase activity (Western blot), and heme oxygenase (HO)-1, induced and protective against OxSt (ELISA).	The study showed that OxSt is activated in ADPKD and that tolvaptan treatment reduces proteins closely related to OxSt signaling, inflammation, and cardiovascular–renal remodeling and helps induce defense against OxSt. In tolvaptan-treated ADPKD patients, the blood creatinine and eGFR levels were 126.3 ± 13.3 µmol/L and 53.8 ± 4.6 mL/min/1.73 m^2^, respectively. In contrast, the blood creatinine and eGFR levels in untreated ADPKD patients were 78.67 ± 11.98 µmol/L and 91.44 ± 14.07 mL/min/1.73 m^2^, respectively.	
	Fujiki et al. [41]	2019	The research used a renal cortical collecting duct cell (mpkCCD) in a cell line and mouse kidneys to determine how tolvaptan and bardoxolone methyl affect Nrf2.	Tolvaptan activated Nrf2 and increased the mRNA and protein expression of antioxidant enzyme heme oxygenase-1 (HO-1) through the phosphorylation of protein kinase RNA-like endoplasmic reticulum kinase.	
BHB	Torres et al. [33]	2019	The research examined the effects of ketogenic diets in adult PKD rats and the effects of BHB treatments in PKD juvenile rats on renal cyst growth in PKD.	BHB affected numerous pathways implicated in PKD, including mTOR, AMPK, and HDACs. BHB-treated rats had a reduced kidney-to-body weight ratio and cystic area compared with the controls.	BHB directly inhibited class 1 histone deacetylases (HDACs), which is theorized to help regulate gene expression by deacetylating lysine residues on histone and nonhistone proteins, leading to changes in gene expression [43]. BHB makes transcription changes in the stress resistance factors FOXO3A and MT2, which promote oxidative stress resistance in the kidneys.
	Shimazu et al. [34]	2013	The research treated human embryonic kidney cells with different amounts of BHB for 8 h to test if BHB has HDAC inhibitor activity, purified and incubated recombinant HDACs to test the inhibitor activity of BHB and its selectivity, and measured the BHB concentration in mouse serum after a 24 h fast to determine if BHB concentrations in vivo affect histone acetylation, all to determine the effectiveness of BHB’s ability to inhibit HDAC.	BHB inhibited HDAC, correlating with changes in gene transcription, including the genes encoding the oxidative stress resistance factors FOXO3A and MT2 through selectively depleting HDAC1 and HDAC2, protecting against oxidative stress.	
SS31	Daneshgar et al. [26]	2021	The research treated ADPKD mice models with SS31 to determine how mitochondrial-targeted antioxidants affect ADPKD progression, and ROS, measuring through hemoglobin, BUN, mitochondrial respiratory complex activity, and LDH activity.	SS31 mitigated the progression of APKD-like disease symptoms in mice, reducing mitochondrial ROS and oxidative damage. Kidney staining showed that SS31 reduced the area of kidney cysts and fibrosis in the experimented mice.	SS-31, as a mitochondria-targeting drug, binds to cardiolipin, assists electron transfer, and limits electron linkage. As a result, it protects the structural integrity of the mitochondria, repairs damaged mitochondria, scavenges ROS, and increases the ATP supply, which reduces oxidative stress and improves apoptosis. Additionally, SS31 scavenges mitochondrial ROS and breaks the oxidative stress cycle, preventing renal tissue in diabetes patients.
	Zhu et al. [44]	2021	The research analyzes the pharmacokinetics of SS31, arguing for possible mechanisms for its protective effects against renal diseases, and examines previous data about SS31’s uses against renal diseases such as animal and cell models such in vivo studies in rats and in vitro studies in mesangial cells to study the how SS31 alleviates kidney disease symptoms	Compared with the placebo, patients in the SS31 group underwent a lower degree of partial tissue hypoxia. Additionally, only SS31 group patients had increased renal blood flow (202 ± 29 to 262 ± 115 mL/min; *p* = 0.04) and renal cortical perfusion (1.99 ± 0.8 to 2.9 ± 1 mL/min/mL) three months after percutaneous renal angioplasty.	
Oxypurinol	Chaudhary et al. [30]	2024	The research measured proinflammatory cytokines, inflammasome, and crystal deposition in the kidneys and the mechanisms of increased cyst growth in PKD mice, PCK rats, and a hepatic disease 1 gene model of autosomal recessive PKD, using a combination of oxonic acid and oxypurinol as a treatment for the PKD mice.	A combination of oxypurinol and oxonic acid significantly reduced the increase in serum uric acid induced by oxonic acid and reduced the kidney weight and the cyst index but did not affect cyst growth in PKD RC/RC mice.	Oxypurinol, in the presence of high concentrations of hypoxanthine and xanthine, binds with xanthine oxidoreductase, inhibiting the hydroxylation of hypoxanthine to xanthine. In turn, it prevents xanthine from converting into uric acid [45].
MitoTEMPO	Sims et al. [46]	2014	The research examined preclinical studies to investigate how rodent models of SAKI can be used as a therapy to restore renal recovery.	MitoTEMPO might be able to target the sources of oxidants, improving mitochondrial function, microcirculatory perfusion, renal function, and long-term survival outcomes. Rodent models have shown promising results in reducing oxidative stress and helping renal function.	Mito-TEMPO is a selective mitochondrial antioxidant attached to a lipophilic triphenylphosphonium cation, which targets it to the mitochondria, promoting mitochondrial function and restoring renal function by targeting oxidants and allowing the renal microcirculation and tubular epithelium time to recover [47].
	Arulkumaran et al. [47]	2021	The research examined the effects of MitoTEMPO ex vivo using adenosine triphosphate and lipopolysaccharide-stimulated rat peritoneal immune cells and rat kidney slices exposed to septic rat serum and used a fluid-resuscitated rat model of sepsis to assess the effects of MitoTEMPO in vivo.	MitoTEMPO decreased septic serum-induced mROS levels and maintained a normal nicotinamide adenine dinucleotide redox state and mitochondrial membrane potential in renal proximal tubular epithelial cells ex vivo. In vivo, compared with the placebo, the Mito-TEMPO group had reduced renal oxidative stress determined by urine isoprostane levels and improved renal dysfunction.	

ADPKD (autosomal dominant polycystic kidney disease), ESKD (end-stage kidney disease), TEMPO (Tolvaptan Efficacy and Safety in Management of Polycystic Kidney Disease and Its Outcomes), REPRISE (Replicating Evidence of Preserved Renal Function: An Investigation of Tolvaptan Safety and Efficacy in Autosomal Dominant Polycystic Kidney Disease), eGFR (estimated glomerular filtration rate), OxSt (oxidative stress), HO-1 (heme oxygenase-1), Nrf2 (nuclear factor erythroid 2-related factor 2), V2 (vasopressin V2 receptor), PKD (polycystic kidney disease), BHB (beta-hydroxybutyrate), HDAC (histone deacetylase), mTOR (mechanistic target of rapamycin), AMPK (AMP-activated protein kinase), FOXO3A (forkhead box O3), MT2 (metallothionein 2), SS31 (elamipretide), ROS (reactive oxygen species), BUN (blood urea nitrogen), LDH (lactate dehydrogenase), ATP (adenosine triphosphate), SAKI (sepsis-associated acute kidney injury), and mROS (mitochondrial reactive oxygen species).

## Data Availability

The data are available in this article.

## Abbreviations

PKD (polycystic kidney disease), CKD (cystic kidney disease), ROS (reactive oxygen species), ADPKD (autosomal dominant polycystic kidney disease), ARPKD (autosomal recessive polycystic kidney disease), NO (nitric oxide), ESRD (end-stage renal disease), NOS (nitric oxide synthase), XOR (xanthine oxidoreductase), PCP (planar cell polarity), cAMP (cyclic adenosine monophosphate), NOX4 (NADPH oxidase-4), NADPH (nicotinamide adenine dinucleotide phosphate), ECs (endothelial cells), TECs (renal tubular epithelial cells), TKV (total kidney volume), GFR (glomerular filtration rate), RAAS (renin–angiotensin–aldosterone system), LVH (left ventricular hypertrophy), BHB (beta-hydroxybutyrate), SAKI (sepsis-induced acute kidney injury), XOis (xanthine oxidase inhibitors), AOPPs (advanced oxidation protein products), UA (uric acid), FRAP (ferric-reducing ability of plasma), ML (machine learning), DL (deep learning), MDA (malondialdehyde), ADMA (asymmetric dimethyl arginine), SOD (superoxide dismutase), and AI (artificial intelligence).
